# Immune reconstitution, glomerulonephritis, and successful treatment with rituximab 

**DOI:** 10.5414/CNCS110061

**Published:** 2020-09-01

**Authors:** George Vasquez-Rios, John C. Edwards, Saketh Tummala, Ashley Chapel, Ramez Sunna, David S. Brink, Christopher Laohathai, Thanh-Mai Vo

**Affiliations:** 1 Internal Medicine Department, Saint Louis University School of Medicine,; 2 Nephrology Division, Department of Medicine, Saint Louis University,; 3 Saint Louis University School of Medicine,; 4 Pathology Department,; 5 Neurology Division, Department of Medicine, Saint Louis University School of Medicine, Saint Louis, MO, USA

**Keywords:** glomerulonephritis, multiple sclerosis, vasculitis, reconstitution syndrome, immune restoration, autoimmunity

## Abstract

Background: Alemtuzumab can induce secondary autoimmunity affecting multiple organs. While kidney involvement is uncommon, it can be associated with devastating forms of glomerulonephritis (GN). Case presentation: A 32-year-old African American woman presented with hypertension, proteinuria, and progressive renal failure. Her medical history was remarkable for secondary progressive multiple sclerosis (SPMS). She had received her first induction dose of alemtuzumab 1 year prior to presentation. Upon evaluation, she had scanning speech, multidirectional nystagmus, and mild edema. Her serum creatinine was 2 mg/dL. Urine studies revealed proteinuria and microscopic hematuria. Her serologic tests were positive for c-antineutrophil cytoplasmic antibodies (> 1 : 640). In addition, she was found to have new-onset severe thyroid dysfunction with antibodies against thyroglobulin and thyroid peroxidase. Kidney biopsy was diagnostic for pauci-immune crescentic GN. The patient was treated with methylprednisolone and rituximab with subsequent renal, thyroid, and neurological recovery. Conclusion: This is an atypical case of GN following therapy with alemtuzumab. We hypothesize that immune reconstitution may be a potential mechanism. Alemtuzumab is a new treatment for SPMS that can be associated with GN. Practice guidelines should address the management of its renal complications.

## Introduction 

Alemtuzumab is a humanized monoclonal antibody with high activity against CD 52 (Campath-1 antigen), a surface protein in lymphocytes with immunomodulatory functions [1]. It induces profound lymphopenia and prolonged alteration of the immune response in patients with multiple sclerosis [2, 3]. During the process of immune recovery, disorganization of the immune response can eventually lead to the generation of self-reactive cells that can predispose to autoimmune conditions with marked organ-specific damage. This phenomenon has been described as “immune reconstitution” and is characterized by an unbalanced humoral response and poor T-cell regulation. This report presents the case of a young woman with secondary progressive multiple sclerosis (SPMS) who developed worsening renal and thyroid function in the setting of immune reconstitution, following the administration of alemtuzumab. 

## Case presentation 

A 32-year-old African American woman was admitted to our institution with subacute progressive renal failure for the past 3 months. Her medical history included tensional headaches and SPMS, which was diagnosed at age 14. She endorsed using sporadic acetaminophen. She failed multiple therapies including interferon β-1b (at age 17) and fingolimod (at age 29). One year prior to admission, she received a cycle of alemtuzumab (12 mg/day for 5 days) with subsequent improvement of her neurological symptoms over the following months. Upon evaluation, her vital signs were as follows: blood pressure 168/95 mmHg, heart rate 77 bpm, respiratory rate 18 rpm, and temperature 36.9 °C (98.5 °F). On examination, she had scanning speech, multidirectional nystagmus, and mild bilateral edema to the ankle. 

Her complete blood count was within normal limits, with no lymphopenia (white blood cell count: 9.4 × 10^9^ cells/mL, lymphocyte %: 16.5). Basic biochemistry was remarkable for creatinine (Cr) at 2 mg/dL (reference range: 0.6 – 1.2 mg/dL, baseline Cr: 0.8 mg/dL), and her blood urea nitrogen (BUN) was 37 mg/dL. Urine studies were relevant for proteinuria (860 mg/24h), hematuria, and leukocyturia with a normal urinary immune-fixation pattern. No red blood cell casts were noted on microscopy. Antinuclear antibodies (ANA, 1 : 320, homogeneous pattern), perinuclear antineutrophil cytoplasmic antibodies (p-ANCA, < 1 : 20), and cytoplasmic antinuclear cytoplasmic antibodies (c-ANCA, > 1 : 640) were detected on the autoimmune work-up. Additional testing included: Smith antibodies: 8 (reference: 0 – 19 units), glomerular basement membrane antibodies: 6 (reference: 0 – 20 units), complement C3: 139 (reference: 82 – 193 mg/dL), complement C4: 44 (reference: 15 – 57 mg/dL), complement CH50: 55 (reference: 40 – 60 U/mL), cryoglobulins (quantitative analysis): negative, and double string DNA antibodies: 24 (reference: 0 – 29 IU/mL). She was noted to have autoimmune hypothyroidism with evidence of antibodies against thyroglobulin (1.9 IU/mL, reference: 0 – 0.9 IU/mL) and antibodies against thyroid peroxidase (43 IU/mL, 0 – 34 IU/mL). Additional studies showed thyroid stimulating hormone (TSH) at 133.15 mIU/mL (reference range: 0.4 – 4.5 mIU/mL), free T3: 1.3 pg/mL (reference range: 1.7 – 3.7 pg/mL) and free T4: 0.5 ng/mL (reference range: 0.7 – 1.5 ng/mL). 

**Figure 1.**
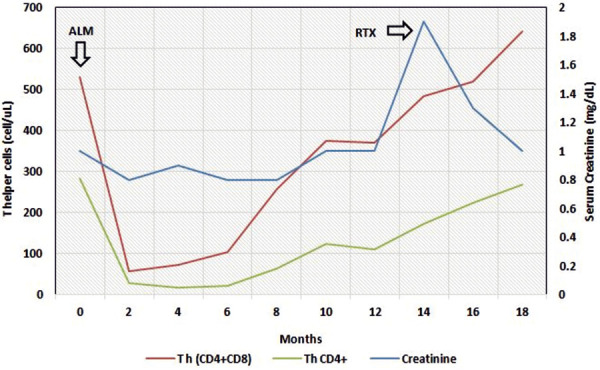
Acute kidney injury (AKI) in the setting of immune reconstitution following treatment with alemtuzumab (ALM). Trajectories of serum creatinine, T-helper (CD4+ CD8+), and T-cell CD4+ counts in relation with drug administration are shown. Rituximab (RTX) was administered during the present admission, and resolution of AKI was seen afterward. High CD8+/CD4+ ratio was noticed throughout the patient’s clinical course.

**Figure 2.**
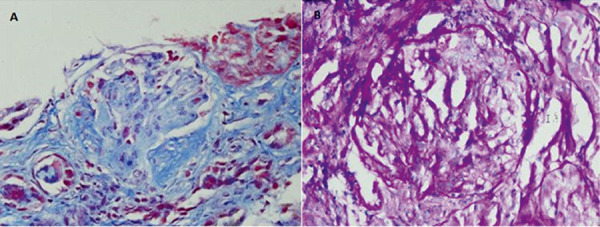
A kidney biopsy showed global glomerulosclerosis in 2 of 14 glomeruli, segmental sclerosis in 1 of 14 glomeruli (A), extra-capillary proliferation (cellular crescents) in 1 of 14 glomeruli (B), glomerulomegaly, ~ 5% tubule atrophy, 10% interstitial fibrosis, and arteriolar hyalinosis. Immunofluorescence analysis showed no significant immunoglobulin or complement label; electron microscopic studies showed nonspecific ultrastructural alterations.

On further review, it was noticed that her thyroid and renal dysfunction followed the slow recovery of her T-helper counts, which occurred ~ 10 months after her initial round of alemtuzumab (Figure 1). Furthermore, a markedly high CD8+/CD4+ ratio was noticed during her recovering process. A kidney biopsy was performed due to the high suspicion of an underlying glomerulonephritis (GN) in the setting of immune reconstitution, which had findings consistent with pauci-immune GN with a focal and segmental lesion (Figure 2). No foot process effacement was noticed on electron microscopy. The patient was initially treated with a 5-day course of IV methylprednisolone (1 mg/kg) and transitioned to rituximab, 500 mg/m^2^/dose, 2 infusions/month, every 6 months for the management of SPMS, concurrent thyroid dysfunction and GN. Her kidney function improved significantly, with a new baseline Cr at 0.9 mg/dL (Figure 1). There was no evidence of proteinuria or hematuria on repeated urinalysis. Her thyroid function gradually improved (TSH: 4.48 mIU/mL, free T4: 1.2 ng/mL). Furthermore, her MS remained controlled with rituximab, with no clinical or radiographic progression at 18 months follow-up. 

## Discussion 

This is the case of a patient who developed acute kidney injury (AKI) and GN concomitantly to the reconstitution of her immune system after a round of alemtuzumab. Although thyroid and platelet dysfunction have been reported in association with this therapy, kidney compromise has been rarely described [9]. While unmeasured genetic and environmental factors may have played a role in the pathogenesis of this condition, a direct correlation between alemtuzumab and the onset of GN and autoimmune hypothyroidism is plausible. Moreover, it is possible that in selected cases, AKI could be one of the most prominent manifestations of a dysregulated immune system in patients exposed to similar immunosuppressive therapies [4]. 

CD52 is a glycoprotein of 12 amino acids anchored to glycosylphosphatidylinositol (GPI). It is widely expressed on the cell surface of immune cells such as mature lymphocytes, natural killer cells, and monocytes/macrophages [5]. T-cells with a high expression of CD52 (CD52^hi^) exert immunoregulatory functions [6]. In diabetes-prone mice of the non-obese diabetic strain, CD52^hi^ were found to suppress T-cell activation and proliferation by releasing soluble CD52 (sCD52). The latter binds the inhibitory receptor sialic acid-binding immunoglobulin-like lectins-10 (Siglec-10), thereby impairing the phosphorylation of the T-cell-receptor-associated kinases Lck and Zap-70 [7]. Alemtuzumab is a monoclonal antibody that targets CD52, thereby inducing profound lymphopenia via complement binding and cell-membrane lysis. Although alemtuzumab has a mean terminal phase half-life of 6.1 days [8], a single course may induce prolonged lymphopenia [1]. For instance, some studies report that recovering CD8+ T-cell and CD4+ T-cell counts to the lower limits of normal range may last up to 20 months and 35 months, respectively [9]. 

Alemtuzumab has been associated with secondary autoimmunity in up to 30% of patients [9]. Although the mechanism is not clear, it is hypothesized to be related to homeostatic proliferation; a process in which T-cell expansion compensates for lymphopenia. This has been shown in animal models, where transferred T-cells tend to be more self-reactive, and less dependent on co-stimulation or antigen burden to be activated when administered to lymphopenic subjects [1]. However, as not everyone with lymphopenia develops autoimmunity, a second “hit” may be required. Recent studies have shown that IL-21 stimulates T-cell turnover thereby increasing the number of opportunities for T cells to encounter self-antigens and break tolerance. It is postulated that this second hit could be related to IL-21 overproduction, which subsequently produces an exaggerated homeostatic proliferation as noted in animal models [1]. Grave’s disease, hypothyroidism, and immune thrombocytopenic purpura are some of the most frequent diseases reported. However, the kidney can also be involved with anti-GBM disease [2, 10, 11] (Table 1) and membranous GN, the latter reported in large clinical trials. 

Among inflammatory demyelinating diseases, such as MS, ANCA positivity occurs in 1 – 7% of cases [12]. A leading hypothesis is that ANCA antibodies induce a proinflammatory state by binding antigens like myeloperoxidase (MPO) and stimulating neutrophils to release reactive oxygen species; subsequently inducing vasculitis [12] This process was thought to require B and T lymphocytes, but more recent data has shown that ANCA plays a more independent role in pauci-immune GN as the anti-MPO function of ANCA alone may be enough to induce GN [1]. While not all MS patients with serum ANCA antibodies develop pauci-immune vasculitis, the presence of ANCAs in MS is associated with worse clinical outcomes, spinal cord involvement, and increased susceptibility to renal disease [1, 12]. 

Glomerulonephritis can develop 9 – 40 months after treatment and may have variable outcomes (Table 1), including dialysis dependence, kidney transplantation, and death [2]. Moreover, alemtuzumab may be more commonly used in upcoming years in cases of severe and refractory MS due to its relatively high effectiveness [1]. This could offer hope to many individuals with severely active disease but will also expose large numbers of patients to the risk of secondary autoimmunity. Therefore, routine screening with urinalysis, renal function panel, thyroid function, and complete blood count may help to identify patients at risk of renal and non-renal complications. In this case, early identification of AKI in the setting of immune restoration may have played a role in the rapid renal function recovery after treatment with rituximab. Teaching points are reflected in Table 2. 

To the best of our knowledge, this is a unique case of pauci-immune GN related to the exposure to alemtuzumab. While this case is limited by the lack of genetic investigations, including high-risk variants of ApoL1 and the presence of anti-phospholipase A1 receptor antibodies, it is possible that immune restoration syndrome was part of the mechanism leading to GN. Attention to these cases is mandatory due to potential adverse outcomes including dialysis dependency and death. Alemtuzumab is a comparatively new treatment for MS, and practice guidelines should address the management of its renal and non-renal complications. Rituximab offers an effective therapy for both MS and GN. 

## Declaration 

Written consent was obtained from the patient for the publication of this manuscript and any accompanying data. 

## Funding 

None.****

## Conflict of interest 

All authors declared no conflict of interest. 

**Table 1.** Summary of cases of alemtuzumab-induced glomerulonephritis reported in the literature.

Author, publication year	Age	Sex	Condition	Total dose of ALM	Renal biopsy findings	Anti-GBM antibodies	Onset of GN (months)*	Outcome
Clatworthy et al. 2008 [10]	40	F	MS	100 mg	Crescentic GN, linear deposition of IgG	Positive	9	Dialysis, renal transplant
Clatworthy et al. 2008 [10]	43	M	AAV	788 mg	Crescentic GN with fibrinoid necrosis, marked linear IgG staining	Positive	10	Dialysis, renal transplant
Meyer et al. 2013 [10]	35	F	RRMS	96 mg	Crescentic GN with necrosis, linear IgG staining	Negative	40	Remission
Meyer et al. 2013 [10]	35	F	MS	160 mg	NA	Positive	10	Dialysis, renal transplant

AAV = ANCA-associated vasculitis; Alemtuzumab = ALM; ANCA = antineutrophil cytoplasmic antibody; GN = glomerulonephritis; MS = multiple sclerosis; NA = non-available information; RRMS = relapsing remitting multiple sclerosis. Search strategy included the following key words: <alemtuzumab> AND <glomerulonephritis>, <alemtuzumab> AND <anti-GBM>; <alemtuzumab> AND <membranous nephropathy>. *Onset of GN after exposure to alemtuzumab.

**Table 2.** Teaching points.

Alemtuzumab is a new treatment for MS that can be associated with severe autoimmunity affecting multiple organs including the kidney in the form of GN.
Kidney damage can present in the setting of immune reconstitution including anti-GMB disease, membranous GN, and crescentic GN.
Outcomes associated to AKI in the setting of reconstitution syndrome are variable, including remission, dialysis dependency, and need for renal transplant. Early identification of these cases is suggested.
Close monitoring with renal function panel, urinalysis, TSH, and complete blood counts may help to identify autoimmune complications in patients exposed to alemtuzumab.
